# Spatial working memory alters the efficacy of input to visual cortex

**DOI:** 10.1038/ncomms15041

**Published:** 2017-04-27

**Authors:** Yaser Merrikhi, Kelsey Clark, Eddy Albarran, Mohammadbagher Parsa, Marc Zirnsak, Tirin Moore, Behrad Noudoost

**Affiliations:** 1 School of Cognitive Sciences (SCS), Institute for Research in Fundamental Sciences (IPM), PO Box 1954851167, Tehran, Iran; 2 Department of Cell Biology and Neuroscience, Montana State University, Bozeman, Montana 59717, USA; 3 Department of Neurobiology Stanford University, Stanford, California 94305, USA; 4 Gianforte School of Computing, Montana State University, Bozeman, Montana 59717, USA; 5 Howard Hughes Medical Institute, Stanford University, Stanford, California 94305, USA

## Abstract

Prefrontal cortex modulates sensory signals in extrastriate visual cortex, in part via its direct projections from the frontal eye field (FEF), an area involved in selective attention. We find that working memory-related activity is a dominant signal within FEF input to visual cortex. Although this signal alone does not evoke spiking responses in areas V4 and MT during memory, the gain of visual responses in these areas increases, and neuronal receptive fields expand and shift towards the remembered location, improving the stimulus representation by neuronal populations. These results provide a basis for enhancing the representation of working memory targets and implicate persistent FEF activity as a basis for the interdependence of working memory and selective attention.

## Abstract (web summary)

Frontal eye field (FEF) is a visual prefrontal area involved in top-down attention. Here the authors report that FEF neurons projecting to V4/MT are persistently active during spatial working memory, and V4/MT neurons show changes in receptive field and gain at the location held in working memory.

Working memory (WM) and attention are two cognitive functions that appear to be conspicuously interdependent and interrelated, both in the context of normal psychophysical performance^1^ and in cognitive dysfunctions^2^,^3^. For example, visual detection and discrimination is improved at memorized spatial locations when compared to other locations^4^, suggesting that the rehearsal of spatial information during WM is sufficient to augment the processing of sensory input at the rehearsed location^5^. This psychophysical evidence is supported by evidence from neuroimaging and neurophysiological studies: modulation of visual cortex has been reported during object-based WM^6^,^7^,^8^,^9^, and via electroencephalogram and functional magnetic resonance imaging (fMRI) measurements during spatial WM^5^,^10^,^11^. More recent evidence suggests that the association between spatial WM and spatial attention is mediated, to some extent, by motor-related signals originating from gaze control structures^12^, suggesting a model in which both the retention of spatial information and the spatially dependent selection of visual information are facilitated by the preparation of gaze commands^13^. What remains unknown, however, is the specific neural circuitry linking attention and WM.

Numerous recent findings point to the frontal eye field (FEF), a gaze control area within prefrontal cortex (PFC), as a source of modulation of visual cortical activity during spatial attention^14^—these include studies using microstimulation, pharmacological manipulations, lesions and neurophysiological measures (reviewed in ref. 15). Owing to its oculomotor activity, the FEF has also been suggested as a source of motor signals driving the presaccadic enhancement or receptive field (RF) changes in posterior visual cortex^16^,^17^. Either or both of these effects could perhaps be mediated via the FEF's direct reciprocal connections with visual cortical areas^18^. We directly studied the signals sent from the FEF to visual cortex and found that persistent, WM-related activity is a predominant property of V4-projecting FEF neurons. Next, we examined how the content of spatial WM affects visual activity within V4 and middle temporal (MT) extrastriate areas. We found that the visual responses of V4 and MT neurons are enhanced at the locus of spatial WM, consistent with a model in which WM signals modulate the gain of visual inputs. The results provide insight into the neural mechanisms by which PFC alters visual representations according to information held in WM, and identifies persistent activity as a source of visual cortical modulation and as a basis for the interdependence of spatial WM and spatial attention.

## Results

### Persistent activity predominates in FEF-V4 projections

**Figure 1:**
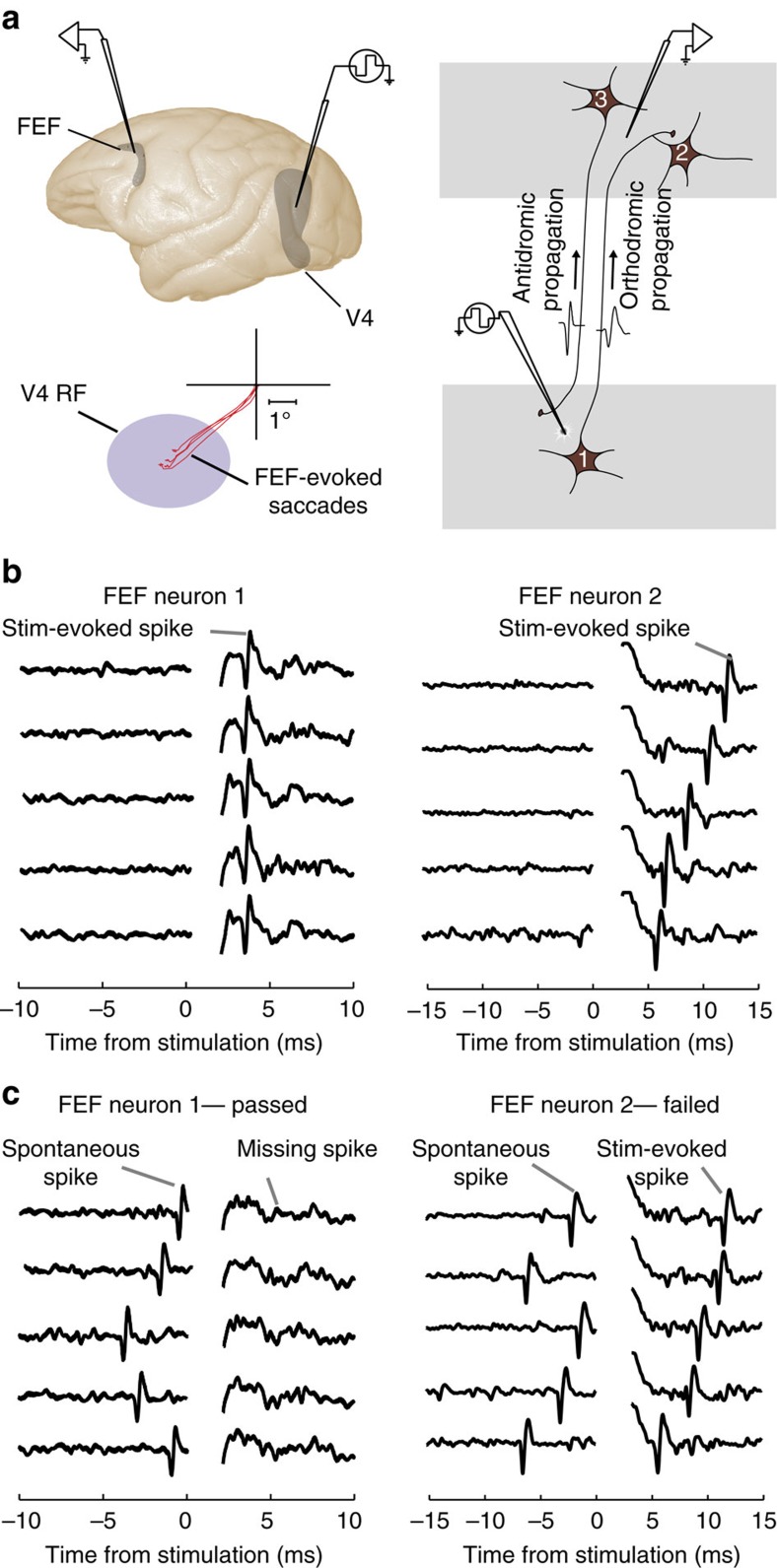
Identification of V4-projecting FEF neurons via antidromic stimulation. (**a**) Simultaneous electrical stimulation of V4 and neurophysiological recording from FEF neurons. We targeted retinotopically corresponding sites within the FEF and area V4 by positioning electrodes in either area such that V4 visual RFs were centred on end points of saccades evoked from the FEF by microstimulation. Right, V4 stimulation could evoke spikes from FEF neurons either via orthodromic or antidromic propagation. (**b**) Five trials recorded from two example FEF neurons that were activated by V4 stimulation. Left, evoked spikes for neuron 1 occurred at a consistent, short latency suggesting antidromic activation. The stimulation-induced artefact near time 0 is omitted for clarity. Right, evoked spikes for neuron 2 appeared at a longer, and more variable, latency, implying a potential synaptic delay due to orthodromic transmission. (**c**) Collision tests for the two example FEF neurons. Left, successful collision test for the first neuron was confirmed by the absence of evoked spikes following spikes occurring spontaneously before V4 stimulation. Right, failure of collision test in the second neuron; V4 stimulation-evoked spikes in this neuron even when delivered shortly after spontaneously occurring spikes.

Anatomical studies show direct projections from the FEF to visual cortex, including areas V4 and MT^18^, yet it is not currently known which of the diverse functional signals of FEF neurons are sent to visual cortex. We therefore assessed the functional properties of FEF neurons identified as projecting to V4. We electrically stimulated V4 while recording FEF neurons with laminar array electrodes and identified V4-projecting FEF neurons using antidromic stimulation and the spike collision test^19^,^20^ (Fig. 1). To accomplish this, we first localized sites within the FEF and V4 where neurons exhibited retinotopically corresponding representations, either in the form of overlapping visual RFs^21^ or V4 RFs that overlapped the end point of saccade vectors evoked by FEF microstimulation^22^. We initially observed that microstimulation of V4 sites evoked spiking activity of FEF neurons only when the end point of the FEF-evoked saccade vector fell within the V4 RF. For these overlapping sites, microstimulation of V4 evoked FEF spikes via both antidromic and orthodromic spike propagation (Fig. 1a). Antidromically activated FEF neurons (neurons passing the collision test) show shorter and more consistent spike latencies, while neurons failing the collision test show longer and more variable latencies (Fig. 1b and Supplementary Fig. 1). V4-projecting, antidromically activated FEF neurons were verified via the spike collision test (Fig. 1b,c; Methods). In this test, when V4 stimulation was delivered within a few milliseconds of a spontaneously generated spike from a recorded FEF neuron, spikes artificially evoked from that neuron by V4 microstimulation were eliminated. Here we discuss the functional characteristics of the antidromically activated, V4-projecting neurons.

**Figure 2:**
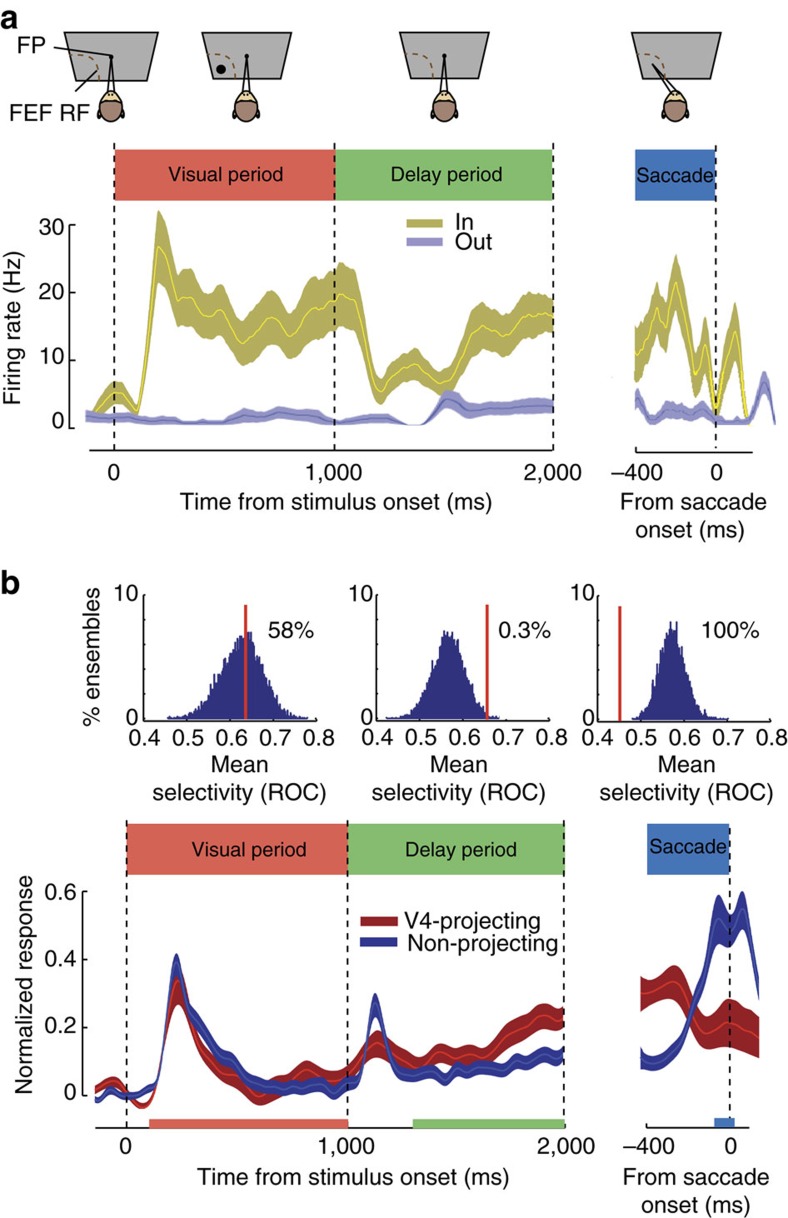
Persistent activity is a predominant property of V4-projecting FEF neurons. (**a**) Response of an example V4-projecting FEF neuron during the spatial WM task in which monkeys made saccades to remembered locations based on a cue at the start of the trial. Neuronal response histogram plots the response of an example FEF neuron on trials when the WM cue appeared in the neuron's RF (yellow) or outside of it (blue). The FEF neuron exhibited elevated activity during both the visual and the delay (memory) periods of the task on trials when the cue appeared in the RF, but activity dropped to baseline before saccade onset. Shaded area indicates ±1 s.e.; FP, fixation point. (**b**) The peristimulus time histogram (bottom) shows the average normalized activity of all V4-projecting FEF neurons (red, *n*=15) versus FEF neurons not projecting to V4 (blue, *n*=307), for trials when the WM cue appeared in the neuron's RF. The histograms (top) show distributions of average selectivity of 1,000 15-neuron ensembles, each selected randomly from the 307 non-projecting FEF neurons. Selectivity was quantified as the ROC for WM in the RF versus outside the RF, separately for the visual, delay and saccade periods. Red vertical lines indicate the average selectivity for the 15 V4-projecting neurons; the location of this mean relative to the distribution of non-projecting ensembles indicates the likelihood that the V4-projecting population differs significantly from the general population in selectivity during this period. Red, green and blue bars along the *x* axis indicate the time window used in analysis for the visual, delay and saccade periods, respectively.

After identifying V4-projecting FEF neurons, we characterized their response properties using an oculomotor, spatial WM task commonly used to differentiate visual, memory and motor components of neuronal activity (see Methods) (Fig. 2a)^23^,^24^,^25^. In this task, the animal must remember the location of a cue throughout a delay period, then saccade to that remembered location to receive a reward. Consistent with previous findings, we found a mixture of visual, memory and motor activity within the overall FEF neuronal population. However, FEF neurons projecting to V4 (*n*=15) exhibited a consistent response profile. Figure 2a shows the response of an example V4-projecting FEF neuron. The neuron responded both to the appearance of the visual cue in its RF and during the memory delay period, but fell silent immediately before saccades into the RF. The neuron did not respond at all during trials in which the cue was presented outside the RF. This response profile was consistent across all 15 FEF neurons identified as V4-projecting. Specifically, only 1 of the 15 V4-projecting FEF neurons exhibited motor activity during the saccade period, and thus the proportion of movement neurons was significantly lower than in the overall FEF population (198 out of 322 neurons overall, *n*_1_=322, *n*_2_=15, *χ*=15.62, *P*<0.001, *χ*^2^ with Yates correction). Furthermore, all of the V4-projecting FEF neurons exhibited significant delay activity during the spatial WM task. In contrast, within the total FEF population, only 54% of neurons (174 out of 322) exhibited significant delay activity. Thus, there was a significantly higher proportion of neurons with delay activity among the V4-projecting FEF population, compared to the population of all FEF neurons (*n*_1_=322, *n*_2_=15, *χ*=10.50, *P*=0.001, *χ*^2^ with Yates correction).

We also compared the magnitude of the selectivity of V4-projecting neurons to that of the overall population of FEF neurons during each task epoch (Fig. 2b). In both the V4-projecting and overall population, we measured the selectivity of neurons for the memory location during the visual, delay and motor periods by contrasting activity when the remembered target was inside the RF to when it was outside of the RF (IN versus OUT). We quantified selectivity using the area under receiver-operating characteristic (ROC) curve, computed from activity within each of the three behavioural epochs (see Methods). We then compared the selectivity of FEF neurons projecting to V4 with that of the overall FEF population during each epoch of the task. To do this, we constructed 10,000 ensembles of 15 neurons by randomly selecting subsets of non-projecting neurons, and compared their average ROC value with that of the V4-projecting population. This analysis revealed that visual period selectivity (visual IN versus OUT) was statistically identical between the two populations (*n*_1_=322, *n*_2_=15; mean ROC: overall=0.63±0.01; V4-projecting=0.64±0.05; *P*=0.924; Wilcoxon rank-sum test). During the visual period, 58% of random non-projecting subsets exhibited greater selectivity than the V4-projecting neurons, reflecting the equal proportion of visually responsive neurons in the V4-projecting and overall FEF populations. Consistent with the disproportionately low frequency of movement neurons in the V4-projecting population, 100% of the random non-projecting subsets had greater movement selectivity than V4-projecting neurons. Accordingly, saccade period selectivity in the overall population was significantly greater than that in the V4-projecting population (*n*_1_=322, *n*_2_=15; mean ROC: overall=0.57±0.01; V4-projecting=0.45±0.03; *P*=0.003; Wilcoxon rank-sum test).

In contrast to what we observed during the visual and saccadic periods, V4-projecting neurons were more selective than non-projecting FEF neurons during the delay period. Delay selectivity for V4-projecting neurons was significantly greater than that of the overall population (*n*_1_=322, *n*_2_=15; mean ROC: overall=0.57±0.01; V4-projecting=0.66±0.05; *P*=0.012; Wilcoxon rank-sum test), consistent with the larger proportion of delay neurons in V4-projecting population. As a result, only 0.3% of the random non-projecting subsets had greater selectivity than V4-projecting neurons. Furthermore, the increase in delay selectivity among V4-projecting neurons grew more pronounced towards the end of the delay period (last 500 ms: *n*_1_=322, *n*_2_=15; mean ROC: overall=0.56±0.01; V4-projecting=0.70±0.03; *P*=0.009; Wilcoxon rank-sum test). Thus, we observed clear differences in magnitude of component signals between V4-projecting neurons and the overall FEF population. Most notably, we found a significant predominance of delay activity being transmitted from the FEF to V4.

### Influence of spatial WM on extrastriate visual responses

Our observation of the predominance of delay activity among V4-projecting neurons may seem surprising given the clear lack of evidence of delay activity in this and other early extrastriate areas^6^,^26^,^27^. Indeed, neurons in such areas are not generally driven by endogenous factors alone. We confirmed that performance during the spatial WM task did not alter the spiking activity of neurons within areas V4 and MT. In the few trials in which no probe was presented during the delay period, neuronal responses were not statistically different during trials in which monkeys remembered targets presented within or outside of the neuronal RF in both area V4 (one-way analysis of variance (ANOVA), main effect of memory location, *F* (3,104)=0.04, *P*=0.989) and MT (one-way ANOVA, main effect of memory location, *F*(3,256)=0.58, *P*=0.629). To more thoroughly verify the absence of changes in baseline firing rate during the delay period, an additional data set was collected, consisting of 90 MT single units in two monkeys recorded during the WM task with no visual probes. The firing rate during the delay period was no different for memory locations inside versus outside the neuronal RF (Supplementary Fig. 2a,b), consistent with previous studies^6^,^26^,^27^. Interestingly, the variability of MT responses decreased during memory of a location inside the RF, despite the lack of changes in firing rate (Supplementary Fig. 2c). Thus, the influence of spatial WM on V4 and MT neurons, and the effect of the memory signal sent from the FEF to V4 (Fig. 2), appeared to be subthreshold.

We sought to reveal the apparently subthreshold influence of WM on V4 and MT neurons. To do this, we used the approach used previously to unmask the ‘silent' inputs to visual neurons from beyond their classical RFs (for example in MT^28^), namely evoking visual responses from neurons with RF probes. We measured the responses of neurons within areas V4 and MT to these probes during the same spatial WM task used to characterize V4-projecting FEF neurons. Probe stimuli were briefly presented (200 ms) at an array of locations (7 × 7) during fixation and during the memory period of the WM task while monkeys remembered targets presented either inside or outside of the neuronal RF (Supplementary Fig. 3). We recorded from single neurons (*n*=92) and multi-unit activity (MUA; *n*=160) in both areas using linear array microelectrodes.

**Figure 3:**
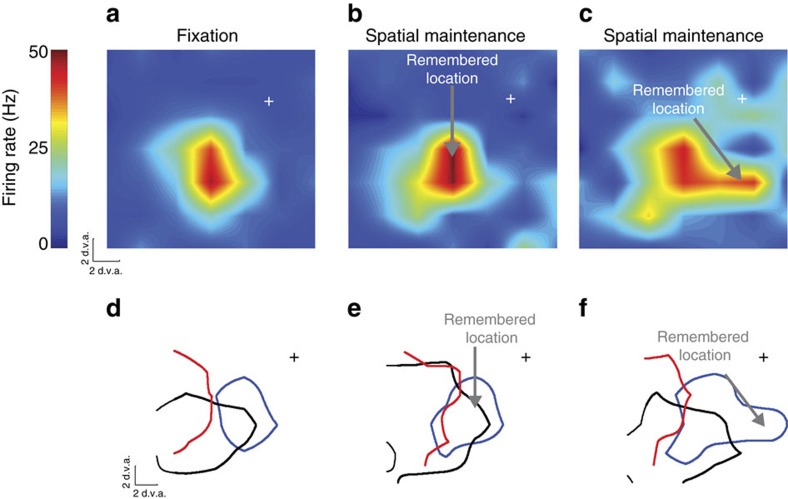
RF changes of area MT neurons during WM. (**a**) Heat map showing the RF of an example MT neuron during fixation (at cross). (**b**) RF of the same neuron measured while the monkey remembered a location inside of the RF, indicated by the arrow. (**c**) RF of the same neuron while the monkey remembered a location to the right of the fixation RF. (**d**) RF outlines of three simultaneously recorded MT neurons during fixation. The blue outline is the RF of the neuron shown in **a**–**c**. Outlines show the 50% response RF boundaries of each neuron. (**e**) Outlines of the same three neurons measured while the monkey remembered a location near the RFs. (**f**) Outlines of the same three neurons measured while the monkey remembered a location to the right of the RFs.

We observed a robust modulation of visually evoked activity within both area V4 and MT that depended on the content of spatial WM. When remembering a location near the fixation RF, the RF of extrastriate neurons, measured by responses to probe stimuli, expanded and shifted towards the remembered location. Changes in the RF of an example neuron from MT are shown in Fig. 3a–c. Contrast the RF of one example neuron during memory maintenance with the same neuron's RF during fixation (Fig. 3a versus Fig. 3b,c): during memory of a location close to the fixation RF (Fig. 3b), the RF expanded by ∼0.5 degrees of visual angle (d.v.a.). However, during memory of a location further from the fixation RF, the RF expanded (2.16 d.v.a.) and shifted (0.57 d.v.a.) towards the remembered location (Fig. 3c). Measured RF perimeters of the same neuron (blue contour) and of two other neurons (black and green) recorded simultaneously during fixation (Fig. 3d) and during memory of two different locations (Fig. 3e,f) exemplify the RF shifts and expansions occurring during the memory period.

**Figure 4:**
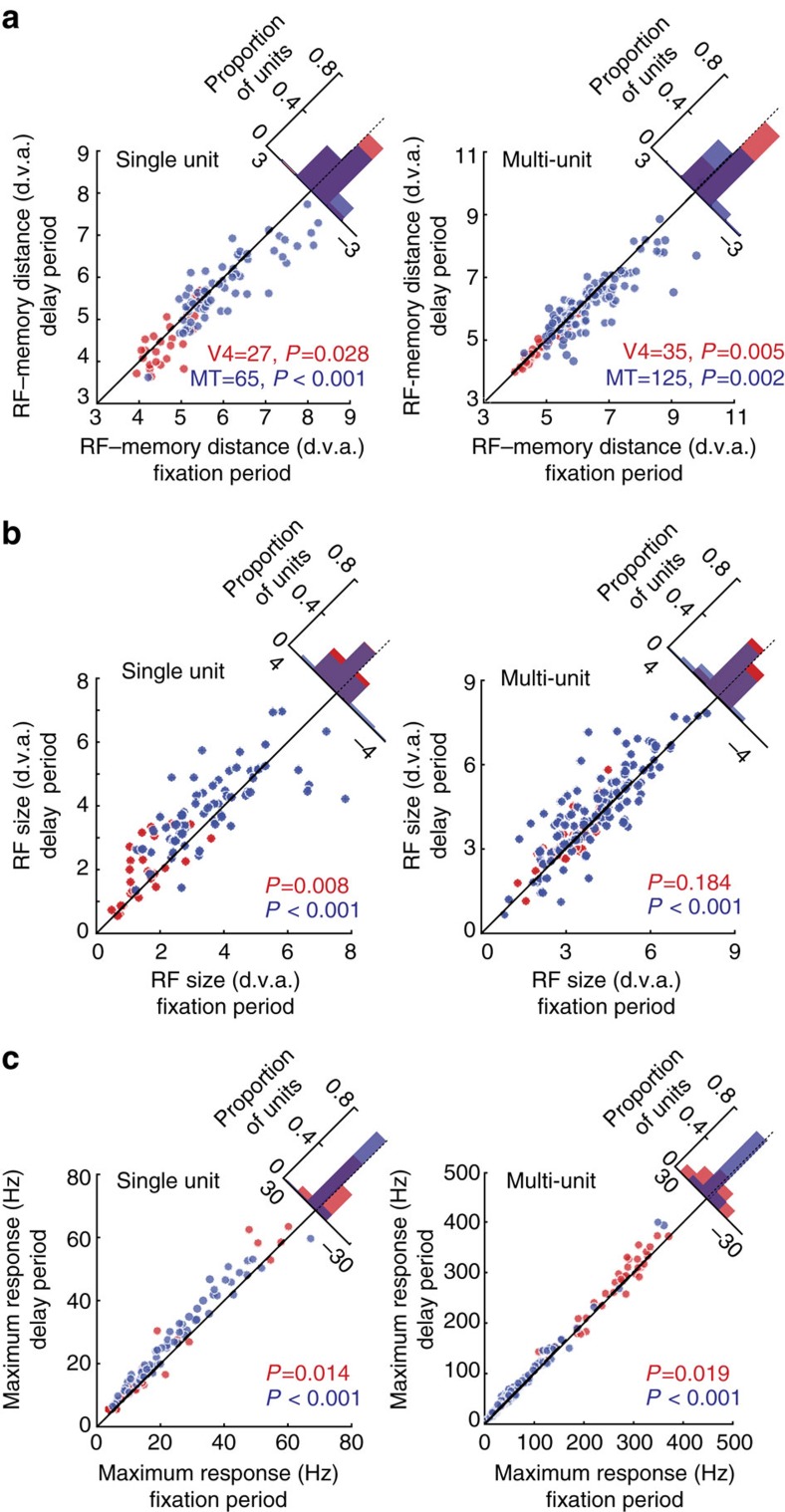
RF changes and gain increases during WM. (**a**) Left: distance between RF centre and the memory location for 27 single neurons in V4 (red) and 65 single neurons in MT (blue), compared between the fixation and delay periods. Histogram in upper right shows the distribution of differences. Right: distance between RF centre and memory location for 35 V4 and 125 MT multi-unit recordings. (**b**) RF sizes compared between fixation and delay periods for single neurons (left) and MUA (right). (**c**) Left: comparison of peak visual responses between fixation and delay periods for single neurons (left) and MUA (right). All *P* values are based on the Wilcoxon sign-rank test.

Figure 4 summarizes the RF changes observed during the WM task for the population of single neurons and for MUA recorded in area V4 and MT. The changes observed in the two extrastriate areas were qualitatively similar and are thus shown together. In both areas, the single-neuron RF centres measured during the memory period had shifted towards the remembered location when compared to RFs measured during fixation (V4: shift=0.21±0.09 d.v.a., *P*=0.028, *n*=27, Wilcoxon sign-rank test; MT: shift=0.26±0.07 d.v.a., *P*<0.001, *n*=65, Wilcoxon sign-rank test; Fig. 4a). A similar decrease in the distance between RF centres and the remembered location was observed for MUA in both V4 and MT (V4: shift=0.07±0.02 d.v.a., *P*=0.005, *n*=35, Wilcoxon sign-rank test; MT: shift=0.20±0.05 d.v.a., *P*=0.002, *n*=125, Wilcoxon sign-rank test). RFs also expanded in size during the memory period, compared to RF size during fixation, for single units in both V4 and MT (V4: expansion=0.44±0.13 d.v.a., *P*=0.008, *n*=27, Wilcoxon sign-rank test; MT: expansion=0.33±0.13 d.v.a., *P*<0.001, *n*=65, Wilcoxon sign-rank test; Fig. 4b). For MUA, this expansion effect only reached significance in MT (V4: expansion=0.14±0.08 d.v.a., *P*=0.184, *n*=35, Wilcoxon sign-rank test; MT: expansion=0.26±0.07 d.v.a., *P*<0.001, *n*=125, Wilcoxon sign-rank test). As a result of these shifts and expansions, more neurons responded to visual stimuli appearing near the remembered target (Supplementary Fig. 4a).

In most cases where visual RFs shift, such as during attention^29^ or before a saccade^30^, the magnitude of the visual response also increases. Indeed, this gain modulation has been proposed as a mechanism driving the shifts in RFs^31^. To determine whether gain modulation of extrastriate visual responses occurred during WM, we compared the peak visual responses to the probes during the memory period to that measured during fixation (Fig. 4c). Peak responses of neurons in both V4 and MT increased during memory of locations near the RF, consistent with an increase in the gain of the visual response. This was true both for isolated single neurons (Δresponse_V4_=1.79±0.77 Hz, *P*=0.014, *n*=27, Wilcoxon sign-rank test; Δresponse_MT_=3.42±0.44 Hz, *P*<0.001, *n*=65, Wilcoxon sign-rank test) and for multi-unit recordings (Δresponse_V4_=7.31±2.80 Hz, *P*=0.019, *n*=35, Wilcoxon sign-rank test; Δresponse_MT_=6.55±0.66 Hz, *P*<0.001, *n*=125, Wilcoxon sign-rank test). When normalized by the average evoked visual response during baseline, spatial WM was associated with a 16% increase in the activity of single units in MT (normalized response_fixation_=2.73±0.11, normalized response_memory_=3.17±0.12, *P*<0.001, *n*=65, Wilcoxon sign-rank test) and a 14% increase in MT MUA (normalized response_fixation_=2.17±0.06, normalized response_memory_=2.47±0.06, *P*<0.001, *n*=125, Wilcoxon sign-rank test). In V4, normalized activity increased by 13% in single units (normalized response_fixation_=5.10±0.58, normalized response_memory_=5.78±0.80, *P*<0.001, *n*=27, Wilcoxon sign-rank test) and by 3% in MUA (normalized response_fixation_=1.87±0.05, normalized response_memory_=1.93±0.05, *P*<0.001, *n*=35, Wilcoxon sign-rank test). The observed changes in visual responses could not be explained by differences in eye position or fixational microsaccades (Supplementary Fig. 5).

**Figure 5:**
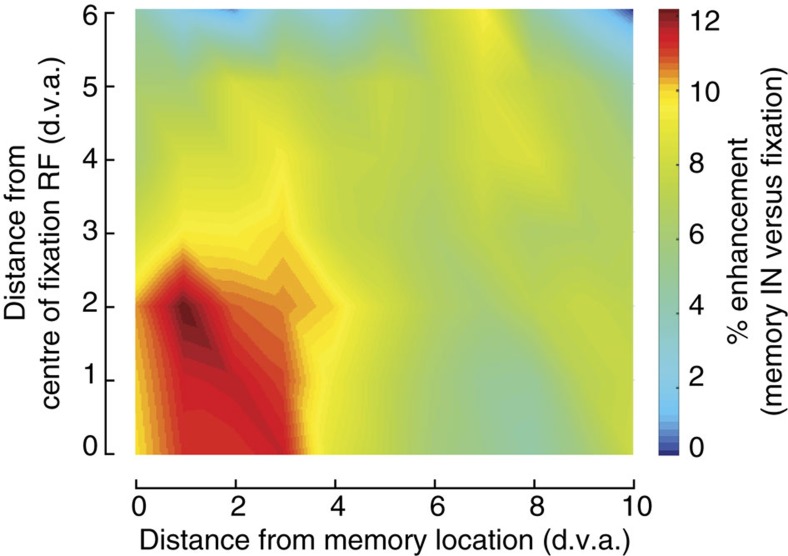
WM-induced changes in extrastriate visual responses improve two-point discrimination. For each neuron and pair of probe positions (*n*=65 MT single neurons and 125 MT MUA), a *d′* value was computed from the responses to two probes to measure two-point discriminability during the fixation and memory conditions. Colour indicates the % enhancement during the memory condition, compared to the fixation condition ((*d′*_memory_−*d′*_fixation_)/*d′*_fixation_ × 100), averaged over all neurons at each location. Axes indicate the distance between the centre of the probe pair and the neuronal RF centre measured during fixation (*y* axis) and the remembered location (*x* axis).

Finally, we tested whether the combined increase in response gain and changes in RF profile improved the population encoding of stimulus position. We compared the ability of neuronal firing rate to differentiate between probe stimuli presented at two different locations during fixation and during memory, equivalent to a two-point discrimination task (see Methods). This analysis revealed that two-point discriminability was enhanced by 12% for the area near the memory and RF locations (Fig. 5). This enhanced discriminability did not depend on the distance between probes (Supplementary Fig. 6), therefore data pooled across probe distances are shown in Fig. 5. This result demonstrates that changes in neuronal responses during memory are beneficial at the level of population representations.

## Discussion

We identified a sample of V4-projecting FEF neurons and found that whereas motor activity was under-represented, memory-related activity was the predominant signal sent from FEF to V4. Memory activity was stronger and more frequent in V4-projecting neurons compared to the overall population. Using the same spatial WM task, we measured neuronal firing rates within area V4 and MT in extrastriate cortex and observed that during the memory delay period, although spontaneous firing rates within these areas were unchanged, V4 and MT visual responses were altered. Specifically, response gain was increased, and RFs expanded and shifted towards the remembered location, resulting in an enhanced representation of targets at the remembered location. Together, these results demonstrate a mechanism by which memory of spatial locations alters the efficacy of visual input. Below, we discuss the relationship of these results to evidence of an interdependence between the mechanisms underlying spatial WM and spatial attention.

**Figure 6:**
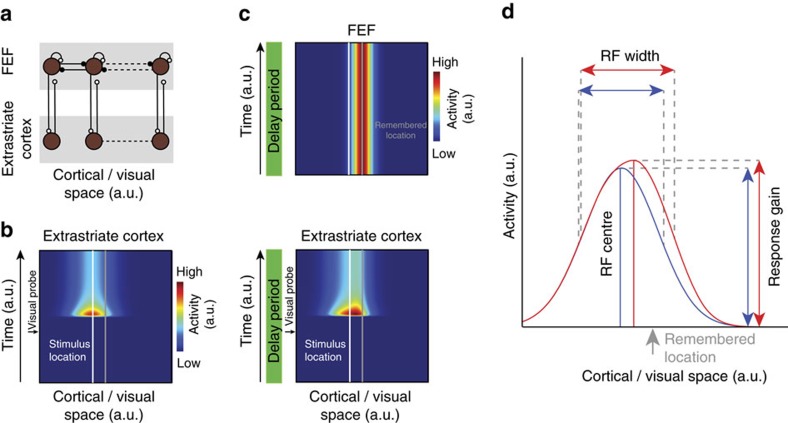
A computational model describing changes in extrastriate visual responses produced by signals from FEF delay neurons. (**a**) Model architecture depicting extrastriate modulation as derived from pools of persistently active, delay neurons in the FEF (brown circles), which are organized topography across visual space (top row). Persistent activity is presumed to emerge from recurrent excitatory connections (white) within each pool^34^,^70^, as well as competitive inhibitory connections (black) between neighbouring pools. Persistent activity is sent via excitatory projections to extrastriate areas such as V4 and MT (bottom row), where neurons project feed-forward inputs to the FEF. (**b**) Population response of model extrastriate units to a visual stimulus presented at a location highlighted by the white line (see Methods for details) in the absence of a memory signal (see Methods for details). (**c**) When remembering a location (grey line), FEF maintains elevated activity (top panel). Consistent with the experimental observations, the delay signal does not alter baseline activity in extrastriate cortex (bottom panel, delay period). However, on presentation of a visual probe at the same location as in **b** the FEF delay signal modulates the response to the visual stimulus when probed during the delay period, resulting in a shift of the population response towards the centre of the delay signal, and an increase in gain. (**d**) Example RF of model extrastriate unit. The blue curve depicts the model RF when measured in the absence of a delay signal. The red curve indicates the RF of the same model neuron when measured in the presence of a delay signal that maintains the location highlighted by the arrow.

Our observation of an increased efficacy of inputs to visual cortex during WM suggests a model whereby persistent, WM-related signals enhance the effective strength of visual inputs. In particular, the results indicate that in such a model, modulation of extrastriate cortical responses by WM-related signals should shift and expand RFs towards remembered locations, enhance visual responses there, yet fail to evoke changes in activity in the absence of visual stimulation. A number of computational models have been formulated to account for changes in visually driven activity and RF dynamics during attention using gain modulation (for example, refs 31, 32, 33). The contributions of a WM-related signal to the enhancement of visual representations can also be explained using a similar framework, one in which gain modulation originates from WM-related, persistent activity^34^,^35^,^36^ from the FEF (Fig. 6). Within this framework, pools of recurrently connected FEF neurons provide the source of persistent delay activity (Fig. 6a). These pools of neurons project to pools of extrastriate neurons in topographic correspondence^37^,^38^. FEF inputs to extrastriate neurons (for example, V4) primarily synapse onto the distal dendritic spines of pyramidal neurons^38^, where their influence on spiking output is expected to be nonlinearly dependent on coincident input^39^,^40^. Modelling the responses of populations of extrastriate units illustrates how such a framework can yield effects consistent with our empirical observations (Fig. 6b,c; see Methods for details). In particular, it shows how spatially specific delay activity from the FEF can bias the population visual response in extrastriate cortex, shifting the population response towards the remembered location. In the presence of this top-down spatial signal, extrastriate units display increased gain, and their RFs expand and shift towards the memorized location (Fig. 6d). Thus, a top-down spatial signal would be sufficient to drive the changes in response gain and RF profile, as observed in our experiments.

Our findings suggest a resolution to a seeming contradiction between existing human fMRI and neurophysiological studies of visual cortical modulation during WM. Psychophysical studies in humans have reported visual perceptual benefits at locations held in WM, an effect resembling that of visual attention^4^. In addition, similar to attention, fMRI studies have reported increased activity within visual cortical areas during WM tasks^5^,^41^. Together this evidence suggests a model in which visual WM involves the recruitment of visual cortical signals^42^,^43^. Yet in spite of this psychophysical and imaging evidence for WM-dependent modulation of visual areas, neurophysiological studies of extrastriate areas like V4 and MT in monkeys have generally found only limited persistent activity in single neurons during the delay period of feature-based WM tasks when visual stimulation is absent^44^,^45^,^46^,^47^. Our results indicate that although neurons in both V4 and MT failed to exhibit changes in firing rate during spatial WM maintenance, they receive a WM-dependent signal during the delay period. This top-down WM signal influences neural activity within these areas only in the presence of visual stimuli. The anatomical evidence that FEF inputs to V4 synapse predominantly on the distal dendritic spines of pyramidal neurons^38^ is consistent with a modulatory role, as opposed to a driving one, of the FEF's delay activity on visual cortical representations. This modulatory role suggests that one reason for the robustness of visual cortical modulation during WM in human studies^8^,^11^,^48^ could be that activity measured by fMRI correlates both with spiking activity as well as synaptic input^49^, and thus WM effects observed in human visual cortex may largely reflect a subthreshold, modulatory influence. Nonetheless, similar to the present results, evidence from studies in humans suggests that PFC, including the FEF, engages visual cortex during WM^50^, and that this engagement may provide a basis for the enhancement of visual cortical representations associated with WM maintenance^42^,^43^. These findings also suggest a specific hypothesis regarding the shared neural mechanisms of prefrontal modulation of visual cortex during attention and WM. Specifically, they suggest that persistent activity sent from FEF to visual cortex is a common mechanism of attention and spatial WM modulation. Indeed, the gain changes and RF shifts reported here for visually evoked responses during WM resemble previously reported effects of attention^31^,^51^,^52^,^53^. Although there is abundant evidence of a contribution of FEF neurons to visual spatial attention and to attentional modulation of visual cortex^21^,^22^,^54^,^55^,^56^, it has remained unclear for some time which class of FEF neurons provides that contribution. Some studies have yielded indirect, correlative evidence of a greater contribution of FEF visual neurons than motor neurons^57^,^58^,^59^, but a direct test of this has been lacking. Our antidromic results directly confirm the absence of a contribution of FEF motor neurons to extrastriate visual cortex. However, rather than a disproportionate input from visual neurons, they show instead that delay neurons, and delay signals, are predominant among V4-projecting neurons.

In addition, previous results show that persistent activity in PFC, the signature of spatial WM, is mediated by dopamine D1Rs^60^. Noudoost and Moore^61^ showed that local pharmacological manipulation of dopamine D1R-mediated activity in the FEF enhances visual signals in V4. Infusion of small volumes of a D1R antagonist, which has been shown to enhance persistent activity in PFC^60^, increases the firing rate, visual selectivity and reliability of responses in V4 neurons with RFs overlapping the FEF infusion site. These results, combined with the present finding that FEF neurons projecting to V4 carry WM-related, persistent activity and that extrastriate responses are enhanced during the maintenance of WM, suggest that the modulatory control of visual cortical signals is specifically achieved by D1R-mediated, persistent activity in the FEF. This could potentially explain how aberrant neuromodulatory control of the neural circuits that link attention and WM may underlie the associated impairments of these functions in several mental illnesses involving an imbalance of prefrontal dopamine^62^.

## Methods

### General and surgical procedures

Four adult male rhesus monkeys (*Macaca mulatta*) were used in this study (two of them for the antidromic experiment and three for the extrastriate recording experiments). All experimental procedures were in accordance with the National Institutes of Health Guide for the Care and Use of Laboratory Animals, the Society for Neuroscience Guidelines and Policies. The protocols for all experimental, surgical and behavioural procedures were approved by the Montana State University Institutional Animal Care and Use Committee. All surgical procedures were carried out under isoflurane anaesthesia and strict aseptic conditions. Before undergoing behavioural training, each animal was implanted with a stainless-steel headpost (Gray Matter Research, Bozeman, MT), attached to the skull using orthopaedic titanium screws and dental acrylic. Following behavioural training, custom-made PEEK recording chambers (interior 22 × 22 mm) were mounted on the skull and affixed with dental acrylic. Within the chambers two 22 × 22 mm craniotomies were performed above the prefrontal and extrastriate visual areas (prefrontal chambers were centred at 42 mm anterior/posterior (A/P), 23 mm medial/lateral (M/L) and 28 mm A/P, 23 mm M/L; extrastriate craniotomies were centred at −6 mm A/P, 23 mm M/L and −13 mm A/P, 23 mm M/L)

### Behavioural monitoring

Animals were seated in a custom-made primate chair, with their head restrained and a tube to deliver juice rewards placed in their mouth. Eye position was monitored with an infrared optical eye-tracking system (EyeLink 1000 Plus Eye Tracker, SR Research Ltd, Ottawa, CA), with a resolution of <0.01° root mean squared; eye position was monitored and stored at 2 KHz. The EyeLink PM-910 Illuminator Module and EyeLink 1000 Plus Camera (SR Research Ltd, Ottawa, CA) were mounted above the monkey's head, and captured eye movements via an angled infrared mirror. Juice was delivered via a syringe pump and the Syringe PumpPro software (NE-450 1L- X2, New Era Pump Systems, Inc., Farmingdale, NY). Stimulus presentation and juice delivery were controlled using custom software, written in MATLAB using the MonkeyLogic toolbox^63^. Visual stimuli were presented on an light-emitting diode-lit monitor (Asus VG248QE: 24in, resolution 1,920 × 1,080, 144 Hz refresh rate), positioned 28.5 cm in front of the animal's eyes. A photodiode (OSRAM Opto Semiconductors, Sunnyvale, CA) was used to record the actual time of stimulus appearance on the monitor, with a continuous signal sampled and stored at 32 KHz.

### Behavioural tasks

Each day began by calibrating the eye position; once the electrode was positioned in the FEF, the same task was used with stimulation to verify that the electrode was in FEF and estimate the RF centre. The fixation point, a ∼1 d.v.a. white circle, appeared in the centre of the screen, and the monkey maintained fixation within a ±1.5 d.v.a. window for 1.5 s. For eye calibration, no stimulation was delivered and the fixation point could appear either centrally or offset by 10 d.v.a. in the vertical or horizontal axis. To establish that the electrode was positioned within the FEF and to estimate the FEF RF location, microstimulation was delivered on 50% of trials; microstimulation consisted of trains (50–100 ms) of biphasic current pulses (≤50 μA; 250 Hz; 0.25 ms duration). On no-stimulation trials, the monkey was rewarded for maintaining fixation; on stimulation trials, the monkey was rewarded whether fixation was maintained or not. The ability to evoke saccades with low stimulation currents (≤50 μA) confirmed that the electrode was in the FEF; the end point of the stimulation-evoked saccades provided an estimate of the RF centre for the FEF site.

Preliminary RF mapping was conducted with a moving bar stimulus. Preliminary RF mapping was conducted by having the monkey fixate within a ±1.5 d.v.a. window around the central fixation point, while ∼2.5 × 4 d.v.a. white bars swept in eight directions (four orientations) across the approximate location of the neuron's RF. Responses from the recording site were monitored audibly and visually by the experimenter, and the approximate boundaries of the RF were noted for the positioning of stimuli in subsequent behavioural tasks.

The memory-guided saccade task with RF mapping was used to assess changes in visual responses during memory. Monkeys were trained to fixate within a ±1.5 d.v.a. window around the central fixation point. After 1 s of fixation, a 1.35 d.v.a. square target was presented and remained onscreen for 1 s. The animal then remembered the target location while maintaining fixation for 1 s (delay period) before the central fixation point was removed. The animal then had 500 ms to move his eyes to a ±4 d.v.a. window around the previous target location, and remain fixating there for 200 ms to receive a reward. RFs of neurons were mapped by presenting brief (200 ms) visual probes (∼1 d.v.a. white circles) in a 7 × 7 d.v.a. grid of locations in 1–2.5 d.v.a. intervals, both before target presentation (baseline RF mapping) and during the delay period (delay period RF mapping). Four probes were presented in succession, with an inter-probe interval of 200 ms. This 7 × 7 grid of probes was positioned to overlap with the RF of the recorded neuron based on the preliminary RF mapping described above. The first probe from each trial was excluded from the analysis. The location of the remembered target could vary with respect to the RF of recorded neurons. On 9% of trials no probes were presented and these trials were used to verify that delay period firing rates within extrastriate areas were not statistically different between different memory conditions.

For the collision experiments, the FEF visual, motor and delay activity were characterized in a separate memory-guided saccade task with no probes. Electrical stimulation was delivered during the fixation, visual, delay or saccade period on 50% of trials (on the other 50% of trials there was no stimulation). Monkeys fixated within a ±1.5 d.v.a. window around the central fixation point. After 1 s of fixation, a 1.35 d.v.a. square target was presented and remained onscreen for 1 s. The animal then remembered the target location while maintaining fixation for 1 s (delay period) before the central fixation point was removed. The animal then had 500 ms to shift its gaze to a ±4 d.v.a. window around the previous target location, and remain fixating there for 200 ms to receive a reward. This task was performed with two potential target locations, located at 0° and 180° relative to the estimated RF centre. For identifying antidromically activated FEF neurons, electrical stimulation consisted of single biphasic current pulses (600–1,000 μA; 0.25 ms duration, positive phase first). Stimulation times were 500 ms after initiating fixation (fixation), 500 ms after visual target onset (visual), 500 ms after target offset (delay) or 150 ms after the go cue (saccade).

### Neurophysiological recording

The electrode was mounted on the recording chamber and positioned within the craniotomy area using a Narishige two-axis platform allowing continuous adjustment of the electrode position. For single-electrode recordings, a 28-gauge guide tube was lowered to contact or just penetrate the dura, using a manual oil hydraulic micromanipulator (Narishige, Tokyo, Japan). Then a varnish-coated tungsten microelectrode (FHC, Bowdoinham, ME), shank diameter 200–250 μm and impedance 0.2–1 MΩ (measured at 1 kHz), was advanced into the brain for the extracellular recording of neuronal activity. Single-electrode recordings used a Plexon pre-amplifier and AM Systems amplifier, filtering from 300 Hz to 5 KHz. For array electrode recordings a 28-gauge guide tube was lowered as described, and the 16-channel linear array electrode (V-probe, Plexon, Inc., Dallas, TX) was advanced into the brain using the hydraulic microdrive. The array electrode was connected to a headstage pre-amplifier (Neuralynx, Inc., Bozeman, MT). Neuralynx Digital Lynx SX and associated software were used for data acquisition. Spike waveforms and continuous data were digitized and stored at 32 kHz for offline spike sorting and data analysis. Areas MT and V4 were identified based on stereotaxic location, position relative to nearby sulci, patterns of grey and white matter, and response properties of units encountered; the FEF was identified based on these factors and the ability to evoke fixed-vector eye movements with low stimulation currents. The location of brain areas within the recording chamber was verified via single-electrode exploration before beginning data collection with the electrode arrays.

### Data analysis

Units without visual responses or defined RFs were excluded. Sample sizes were based on the number of neurons commonly reported in previous literature. Trial order was randomized, with comparisons generally occurring within a specific neuron between conditions. No blinding was used. Most statistical comparisons used non-parametric tests.

The visual, motor and delay period activity of FEF neurons were measured using the spatial WM task described above. The visual period included activity 100–1,000 ms after stimulus onset. Delay period activity was measured from 300 to 1,000 ms after stimulus offset. Motor activity was quantified in the perisaccadic window from 75 ms before to 25 ms after the saccade onset. These time windows were also used for the ROC selectivity analysis described below. When determining whether a neuron had significant visual or delay activity, activity in the visual and delay periods of the IN condition was compared to the activity of the same neuron during fixation (300 ms before stimulus onset), using the Wilcoxon sign-rank test (*P*<0.05). When determining whether a neuron had significant motor activity, saccade-aligned activity in the IN condition was compared to saccade-aligned activity earlier in the trial (450–250 ms before saccade onset), using the sign-rank test (*P*<0.05).

The strength of the selectivity of neurons during the visual, delay and motor epochs was quantified using the ROC method to compare the distribution of firing rates for trials in which the WM cue appeared inside versus outside the neuron's RF^64^. The areas under ROC curves were used as a measure of selectivity for cue location, and were calculated as in previous studies^65^,^66^. Specifically, we computed the average firing rate in the visual, delay and saccade windows defined above, for cue in and cue out trials. We then computed the probability that the firing rate in each stimulus condition exceeded a criterion. The criterion was incremented from 0 to the maximum firing rate, and the probability of exceeding each criterion was computed. Thus, a single point on the ROC curve is produced for each increment in the criterion, and the entire ROC curve is generated from all of the criteria. The area under the ROC curve is a normalized measure of the separation between the two firing rate distributions obtained when the WM cue appeared inside versus outside the neuronal RF, and provides a measure of how well the neuronal response discriminates between the two conditions.

For the FEF-normalized population peristimulus time histogram plots shown in Fig. 2b, to compare responses between different populations of neurons in different task periods, the responses of individual neurons were normalized between peak and baseline (FR−baseline)/(peak−baseline). For the MT-normalized population peristimulus time histogram plots shown in Supplementary Fig. 2, where the comparison was between conditions within each neuron; the responses of individual neurons were normalized by their mean during the visual period.

Fano factor (Supplementary Fig. 2c) was computed as the variance/mean of neuronal responses in a single condition across multiple trials, using the mean matching methods developed by Churchland *et al*.^67^. In outline, this method involves looking at the firing rate for each neuron and time bin, and discarding points until a common firing rate distribution between conditions is achieved. Rates were computed in a sliding 100 ms bin spanning the delay period analysis window.

For RF mapping, neuronal responses to the probes were measured in the window 30–160 ms after probe onset. RF contours were defined as the area of the visual field with a response more than 0.75 × (maximum–minimum visual response) of that unit. The RF centre was defined based on the centre of mass of this RF area. The RF size was defined as two times the square root of the area divided by pi (an approximation of RF ‘diameter'). For the response gain and RF calculations, the peak response in each of the three in-hemifield memory conditions was averaged together.

We examined our findings for any influence of eye position or microsaccades. During the spatial WM task, eye position showed no systematic biases within the fixation window based on task epoch or memory location (Δeye position, memory in versus fixation=0.02±0.01 d.v.a., *P*=0.153, *n*=22, Wilcoxon sign-rank test). For RF mapping and the measurements in Figs 3 and 4, the eye position was corrected based on the actual eye position within the fixation window at the moment of probe presentation, using the eye monitoring and photodiode information. The gain effects shown in Fig. 4c were independent of small deviations in eye position (Supplementary Fig. 5a). Unlike mean eye position, microsaccade properties did vary between the fixation and memory periods; however, these changes in microsaccades were not related to the gain effects. Microsaccade direction was biased towards the remembered location during the memory period compared to the fixation period (distance from the remembered location, start versus end of microsaccade, Δ_memory–fixation_=0.06±0.01 d.v.a., *P*<0.001, *n*=22, Wilcoxon sign-rank test), but there was no effect on gain (ANOVA, *F*(98,1484)=0.187, *P*=1). Microsaccade amplitude was larger during the fixation period than the memory period (Δmicrosaccade amplitude=0.07±0.01 d.v.a., *P*<0.001, *n*=22, Wilcoxon sign-rank test; microsaccade amplitude_fixation_=0.44±0.03 d.v.a., *P*<0.001, *n*=22, Wilcoxon sign-rank test; microsaccade amplitude_memory_=0.38±0.02 d.v.a., *P*<0.001, *n*=22, Wilcoxon sign-rank test). Again, however, there was no relationship between microsaccade amplitude and gain (ANOVA, *F*(91,1573)=0.971, *P*=0.734). Microsaccade velocity was greater during the fixation period than the memory period (Δmicrosaccade velocity=2.15±0.38 d.v.a. s^−1^, *P*<0.001, *n*=22, Wilcoxon sign-rank test; microsaccade velocity_fixation_=24.63±0.89 d.v.a.s^−1^, *P*<0.001, *n*=22, Wilcoxon sign-rank test, microsaccade velocity_memory_=22.48±0.79 d.v.a.s^−1^, *P*<0.001, *n*=22, Wilcoxon sign-rank test), but this velocity was unrelated to gain changes (ANOVA, *F*(69,1004)=0.390, *P*=1). Although microsaccade properties differed between memory and fixation conditions, gain enhancement did not depend on microsaccade direction, amplitude or velocity (Supplementary Fig. 5b–d).

To quantify the two-point discrimination between two probes as used in Fig. 5a, we used the *d′* index, defined as


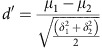


where *μ*_1_ and *μ*_2_ are average responses to each probe and *δ*_1_ and *δ*_2_ are s.d.'s of responses to those two probes. Figure 5a is generated by averaging the *d′* values in 5 d.v.a. bins on each axis. For example, the value assigned to point 0 and 0 is the *d′* modulation for pairs of probes that their distance from memory location and distance from the fixation RF centre were both between 0 and 5 d.v.a.

To model the observed changes of extrastriate visual signals during the delay period of our WM task we adapted a computational framework of attention developed by Hamker^68^,^69^. At the heart of this framework are competitive dynamics between pools of neurons due to gain control and inhibitory mechanisms. In the following, we provide a brief mathematical summary of our simulations. For an in-depth description of the framework please refer to the original studies.

We simulated a pool of gain-modulated extrastriate neurons with RF centres at locations 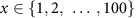. The change of activity 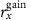
over time is given by


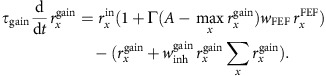


with 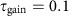, 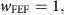 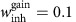 and *A*=1, which shuts down the gain control of the stimulus-driven input activity 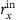 with 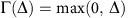. The stimulus-driven activity over time is given by 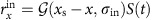, with 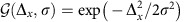, 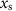 denoting the stimulus location and 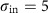. The function 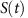 matches the typical stimulus-evoked response of visual neurons and is set to zero otherwise. The FEF delay activity was assumed to be constant over time and follows 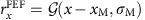 with 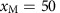 denoting the memorized location and 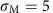. At this stage, the model dynamics lead to a simple up- or downregulation of the firing rate of neurons, dependent on their topographic correspondence with the FEF projections. Whereas these dynamics can distort the population response towards the memorized location as shown in Fig. 6c, they do not alter the RF of neurons at this stage. However, RF changes can be observed for neurons that are driven by those distorted population responses^44^,^45^. To demonstrate this, we simulated another pool of neurons with RF centres at locations 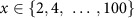, which are simply pooling the activity of the gain stage. The change of activity 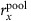
over time is given by


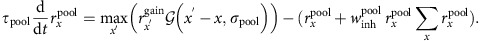


with 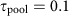, 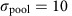 and 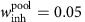. An example RF of a pool neuron is shown in Fig. 6d. Consistent with our empirical observations this RF expands and shifts closer towards the memorized location during the delay period and exhibits an increase in the peak firing rate as well.

### Data availability

The data sets generated and analysed for the current study are available from the corresponding author on reasonable request. MATLAB code used for analysis is available from the corresponding author on request.

## Additional information

**How to cite this article:** Merrikhi, Y. *et al*. Spatial working memory alters the efficacy of Input to visual cortex. *Nat. Commun.* **8**, 15041 doi: 10.1038/ncomms15041 (2017).

**Publisher's note:** Springer Nature remains neutral with regard to jurisdictional claims in published maps and institutional affiliations.

## Supplementary Material

Supplementary Information Supplementary Figures
