# Supplementary material for: Spatial working memory alters the efficacy of input to visual cortex
Source: Nat Commun. 2017 Apr 27;8:15041. doi: 10.1038/ncomms15041 (PMC5414175; doi:10.1038/ncomms15041)
Supplement: Supplementary Information — Supplementary Figures [file ncomms15041-s1.pdf]

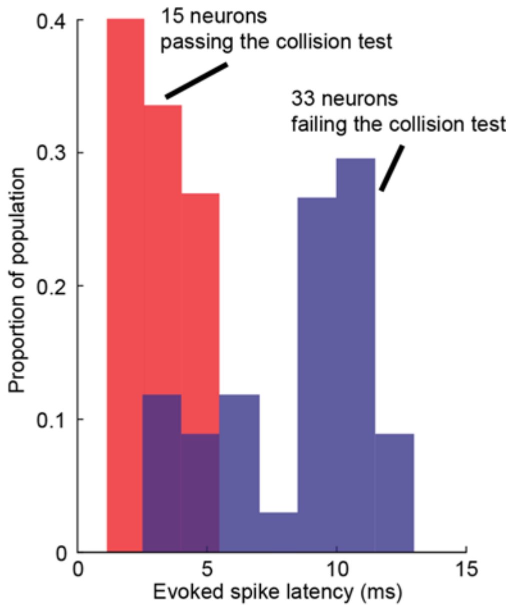

Supplementary Figure 1

Latency of FEF neurons passing (antidromically activated from V4) and failing the collision test. The latencies of evoked spikes for neurons passing the collision test (classified as antidromically activated) was significantly shorter (*n*=15, red) than the latency of neurons failing the collision test (*n*=33, blue; mean $latency_{passing\ collision}$=2.75 ± 1.36 ms, mean $latency_{failing\ collision}$=8.66 ± 0.08 ms, Wilcoxon rank-sum, *P*<0.001). Although the distribution of latencies for neurons failing the collision test appeared bimodal, the bimodality did not reach statistical significance (Hartigan's dip test, *n*=48, *P*=0.140). The antidromic neurons are those shown in figure 2.

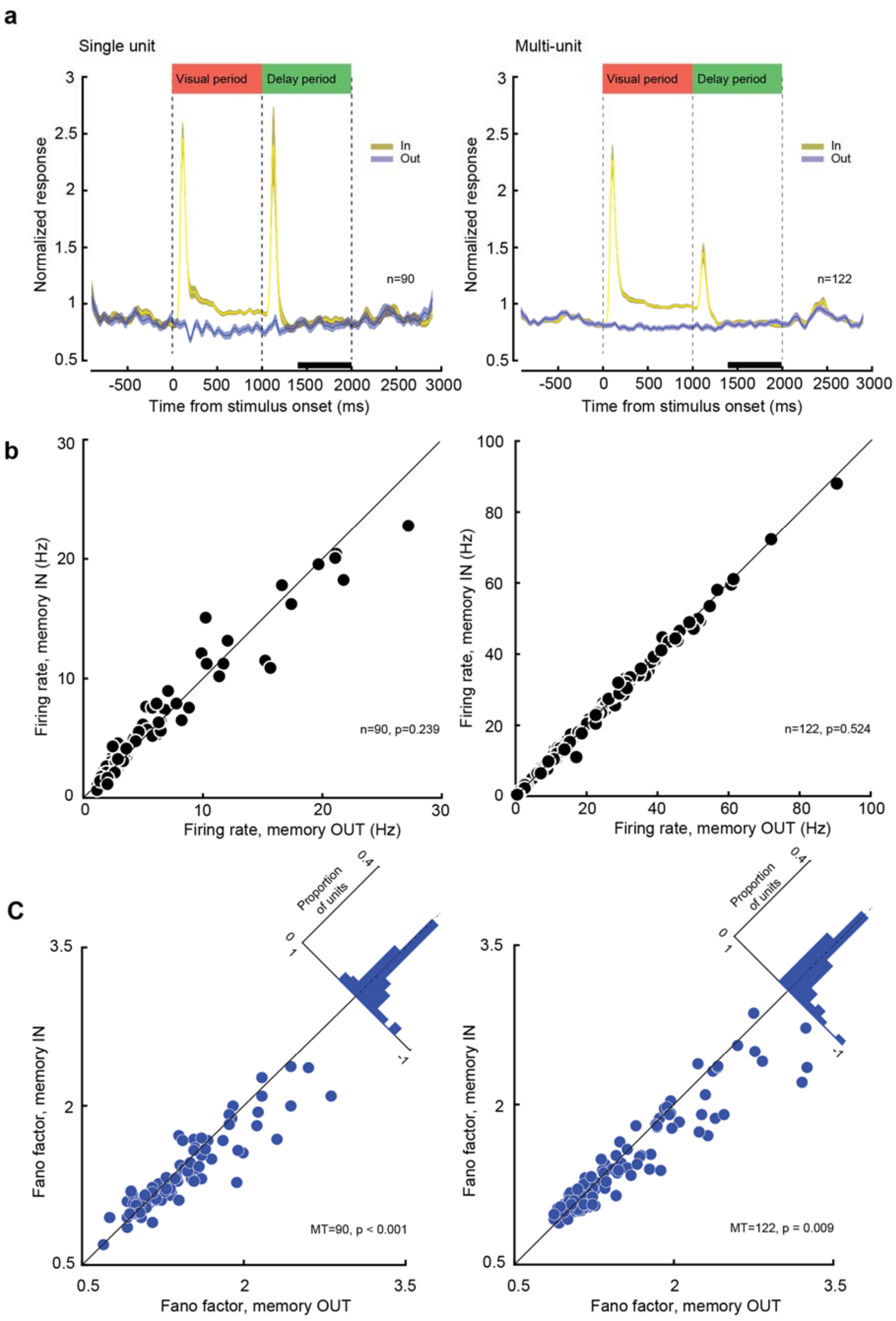

a
Single unit
Visual period
Delay period
In
Out
n=90
Normalized response
Time from stimulus onset (ms)
Multi-unit
n=122
b
Firing rate, memory IN (Hz)
Firing rate, memory OUT (Hz)
n=90, p=0.239
n=122, p=0.524
C
Proportion of units
Fano factor, memory IN
Fano factor, memory OUT
MT=90, p < 0.001
MT=122, p = 0.009

Supplementary Figure 2

Delay period activity in MT does not change based on the location held in memory, but variability decreases when remembering a location in the RF. (a) PSTH of the MT response during the WM task with no probes during the delay period, for stimuli in the RF (yellow) or in the opposite visual hemifield (blue). These neurons are from a separate dataset from the main figures, comprised of 90 single neurons (left) and 122 multi-units (right). The dark bar from 400-1000ms after the offset of the visual stimulus indicates the period of the delay period used for the analysis in parts b-c of this figure. (b) A scatter plot comparing the activity of MT neurons during the delay period for memory locations inside vs. outside the RF. There is no significant change in MT firing rate based on memory location for either single neurons or MUA. (c) The variability of single-neuron and multi-unit MT responses decreases when remembering a location in the RF. Scatter plots compare the Fano factor during the delay period, for memory locations inside vs. outside the RF. The Fano factor is significantly lower when remembering a location in the RF, both for single neurons and MUA. All p-values are from a Wilcoxon sign-rank test.

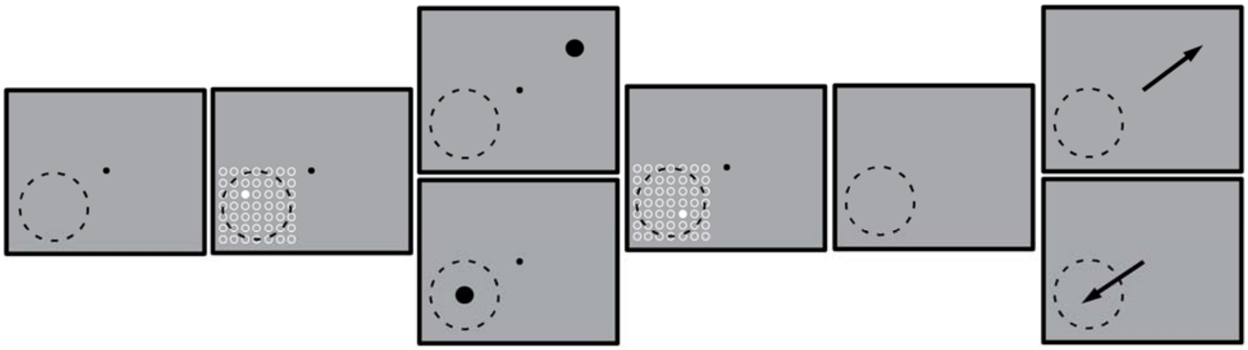

Supplementary Figure 3

Fixation and memory period RF mapping paradigm. The monkey initiated a trial by fixating the central fixation point. The approximate RF of the neuron being recorded, mapped using moving bars prior to this task, is shown by the dashed circle. During 1 s of continued fixation, the baseline RF was mapped by a pseudo-random sequence of probes flashed for 200 ms in a 7x7 grid of potential locations. The WM task target was then presented for 1 s. Although only two potential memory target locations are shown, experiments were carried out with four potential target locations, three in the same hemifield as the RF of the neuron being recorded, and one in the opposite hemifield. During a 1 s delay period, the RF of the neuron was mapped again. The disappearance of the fixation point served as the go cue, and the monkey was rewarded for a saccade to the target's original location.

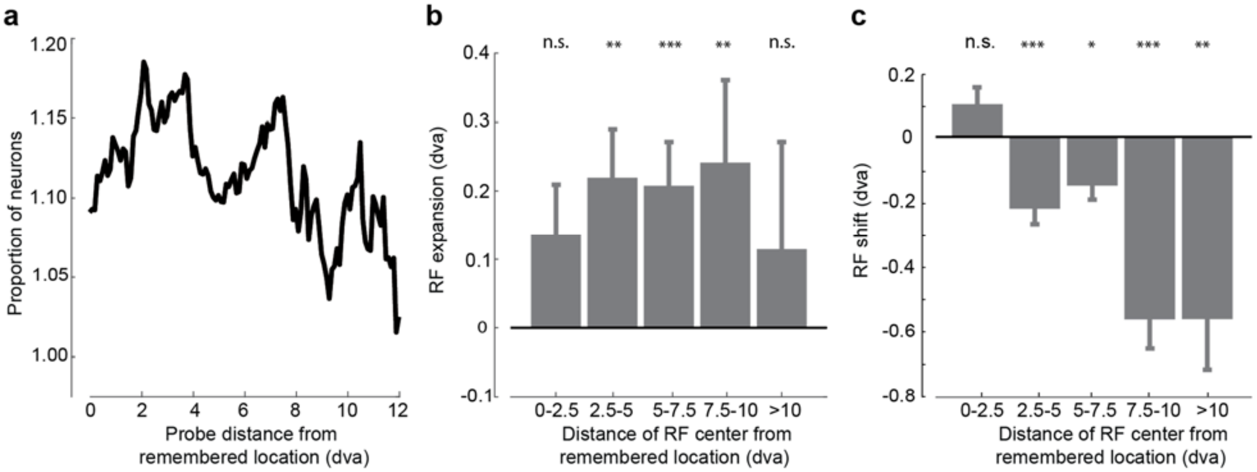

Supplementary Figure 4

Changes in RFs as a function of distance from remembered location. (a) Memory maintenance increases the number of neurons responsive to probes. Plot shows the proportion of neurons (memory IN/OUT) responsive to probes at different distances from the remembered location. The proportion was the ratio of the number of neurons (multi + single units) responsive to the probe above a threshold (0.75*(maximum-minimum visual response)) while the monkey remembered one of three locations in the same hemifield divided by the number of neurons responsive while the monkey remembered a location in the opposite hemifield. (b) RF expansion as a function of the distance between the RF center and the remembered location. Here and in C, each unit contributed 3 values based on the distances for the 3 different in-hemifield memory conditions. The expansion was most pronounced for intermediate distances. (c) RF shifts as a function of the distance between the RF center and the remembered location. Shift values increased as the distance between the RF and the remembered location increased. n.s. $P>0.050$; * $P<0.050$; ** $P<0.010$; *** $P<0.001$, Wilcoxon sign-rank test.

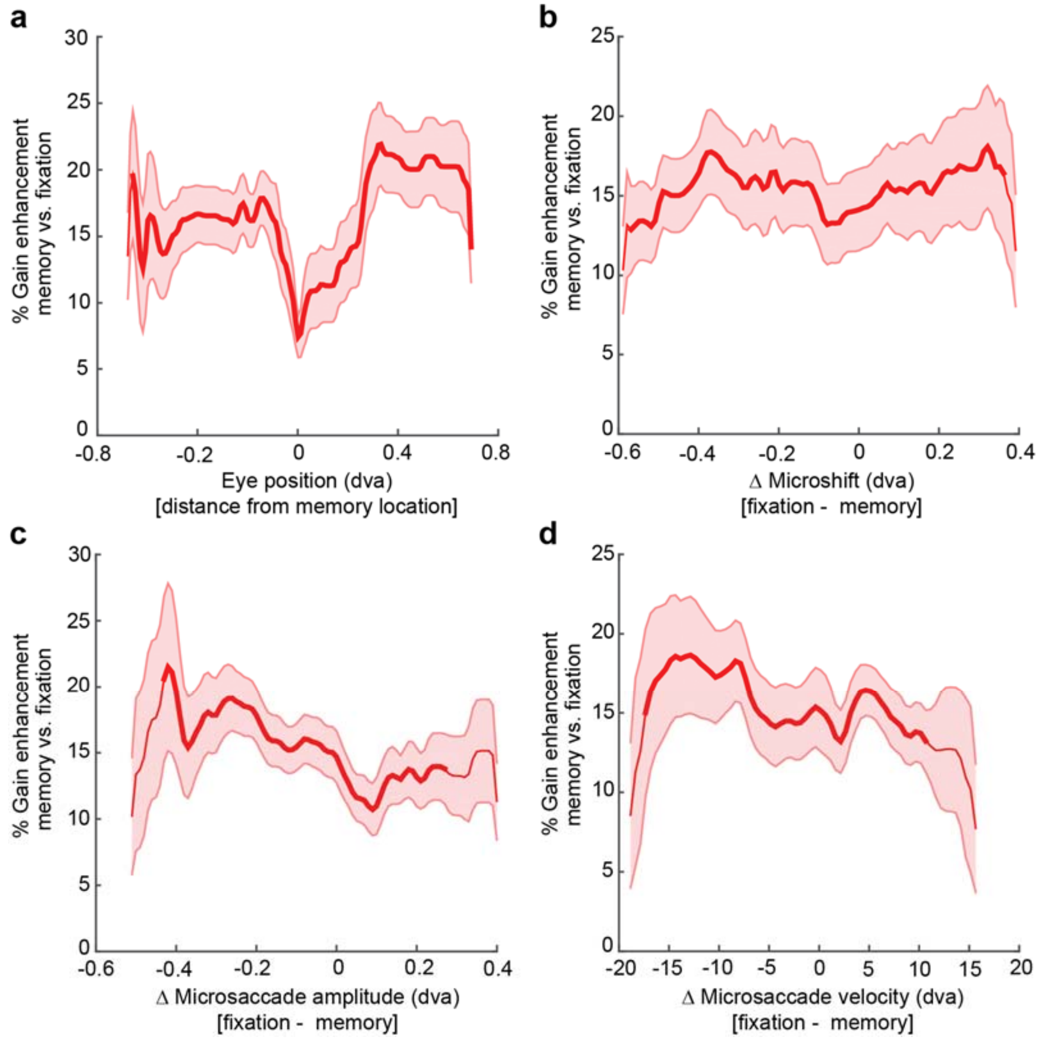

Supplementary Figure 5

Gain enhancement during memory could not be explained by deviations in eye position or microsaccades. The changes in peak response shown in Fig. 4C did not depend upon changes in true eye position or microsaccades within the fixation window. There was no change in average eye position based on memory condition (Δeye position, memory in vs. fixation=0.02 ± 0.01 dva, *P*=0.153, *n*=22, Wilcoxon sign-rank test). (a) Plot shows gain enhancement as a function of true eye position at the time of probe presentation. Negative values on the x-axis correspond to an eye position closer to the memory location. Gain enhancement was significant throughout the range of actual eye

positions, and whether the eye was shifted toward or away from the memory location relative to the fixation point. An ANOVA showed no effect of eye position on gain (one-way ANOVA, $F(137,2479)=0.792$, $P=0.962$). Bold portions of the line show significant gain enhancement ($P<0.050$, Wilcoxon sign-rank test). (b) Gain enhancement as a function of microsaccades toward the remembered location at the time of probe presentation. 'Microshift' refers to the difference in the distance from the remembered location at the start vs. the end point of a microsaccade; negative values on the x-axis indicate larger shifts toward the remembered location during the memory compared to the fixation period. Gain enhancement was significant throughout the range of microsaccade values, and an ANOVA showed no effect of microsaccades on gain (one-way ANOVA, $F(98,1484)=0.187$, $P=1$). (c) Gain enhancement as a function of the amplitude of microsaccades at the time of probe presentation. Negative values on the x-axis indicate larger microsaccades during the fixation compared to the memory period. Gain enhancement was significant throughout the range of amplitude values, and an ANOVA showed no effect of microsaccade amplitude on gain (one-way ANOVA, $F(91,1573)=0.971$, $P=0.734$). (d) Gain enhancement as a function of the velocity of microsaccades at the time of probe presentation. Negative values on the x-axis indicate faster microsaccades during fixation compared to memory period. Gain enhancement was significant throughout the range of velocity values, and an ANOVA showed no effect of microsaccade velocity on gain (one-way ANOVA, $F(69,1004)=0.390$, $P=1$).

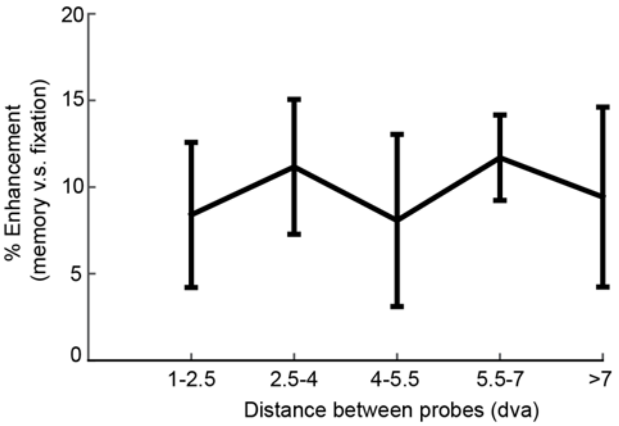

Supplementary Figure 6

Improvement in 2-point discriminability during memory did not depend upon distance between the probes. For the same 190 MT neurons used in figure 5a, this plot shows the improvement in *d'* during the memory condition ($(d'_{memory} - d'_{fixation}) / d'_{fixation}$) as the distance between the two probes varies. There was no significant effect of the distance between probes on the improvement in *d´* (one-way ANOVA, $F(4,935)=0.580$, $P=0.678$). This analysis serves as a control for the enhancement shown in figure 5a, thus focused on pair of probes with their distance from memory location≤8 dva and distance from fixation RF≤7 dva).
